# An interim analysis of the standard care (stoma bags) and double-diapers in pediatric patients with stomas at a tertiary hospital in South Africa

**DOI:** 10.1007/s00383-026-06527-y

**Published:** 2026-07-03

**Authors:** Stephanie Karen Van Straten, Juan Scribante, Amy Botes, Ra’ees De Witt, Coenrad Hendrik Lamprecht, Andonia Papavarnava, Hester Gertruida Oelofse, Cyle Rockman, Catterina Bebington, Dhashna Bloem, Lindiwe Dyamara, Giulia Brisighelli

**Affiliations:** 1 Department of Surgery, School of Clinical Medicine, Faculty of Health Sciences, University of the Witwatersrand, Johannesburg, South Africa; 2 Department of Paediatric Surgery, School of Clinical Medicine, Faculty of Health Sciences, University of the Witwatersrand, Johannesburg, South Africa; 3 Unit for Undergraduate Medical Education, Faculty of Health Sciences, University of the Witwatersrand, Johannesburg, South Africa; 4 Johannesburg Paediatric Colorectal Clinic, Chris Hani Baragwanath Academic Hospital, Soweto, South Africa; 5 Surgeons for Little Lives, Johannesburg, South Africa; 6 Department of General Surgery, Chris Hani Baragwanath Academic Hospital, 26 Chris Hani Road, Diepkloof Soweto, Johannesburg, 1860 South Africa

**Keywords:** Double-diaper, Stoma, Colostomy, Ostomy, Pediatric

## Abstract

**Purpose:**

In resource-limited settings, commercial stoma bags are often unavailable for pediatric colostomy patients, necessitating affordable and accessible alternatives for colostomy care. This is an interim analysis of a study comparing standard of care (stoma bags (SB)) with double-diaper (DD) in patients with colostomies at the Johannesburg Pediatric Colorectal Clinic (JPCC) at Chris Hani Baragwanath Academic Hospital, South Africa.

**Methods:**

A prospective, randomized descriptive study enrolled children < 3 years with newly created colostomies whose caregivers consented; participants were randomized via REDCap to SB or DD and followed up at routine visits until colostomy closure.

**Results:**

Of 45 screened patients, 40 were enrolled; 22 (55%) were female, with a mean (SD) age of 110.15 (151.22) days and weight of 4.57 (1.51) kg. Most had anorectal malformations (30, 75%), end colostomies (24, 60%), and 29 (72.5%) had sigmoid colostomies. At analysis, 23 (58.97%) underwent closure. At enrolment, 19 (47.50%) were randomized to SB and 21 (52.50%) to DD; 28 (71.79%) remained in their assigned group, while 12 (29.27%) crossed over due to guardian preference (7, 38.89%) or healthcare provider recommendation (11, 61.11%). Baseline mean (SD) peristomal skin scores (using the OST scoring system) were 0.83 (1.99) for the SB group compared to 2.31 (3.61) (worse) for the DD group (*p* = 0.402). Follow-up scores among those remaining in their groups were: SB group 0.55 (1.54) and DD group 1.7 (2.51) (*p* = 0.002). Patients who crossed over had mean (SD) scores of 1.42 (2.46) at enrollment and 2.05 (2.63) at follow-up. Mean (SD) clinic visits were 3.92 (2.47) for the SB group, 5.73 (3.87) for the DD group, and 7.75 (2.83) for the mixed group (*p* = 0.01). When supplies were depleted, 12 (28.57%) returned for more of their respective product, while 29 (69.05%) purchased diapers. Care givers out-of-pocket expenditure as lower in SB 1 (6.7%) and 5 (29.4%) in DD group (*p* = 0.01). Patients who remained in the SB group reported satisfaction with bag quality 10 (66.7%), compared to those who crossed over 4 (26.7%) (*p* = 0.02).

**Conclusion:**

Diapers are an accessible alternative to commercial stoma bags for patients with colostomies.

**Supplementary Information:**

The online version contains supplementary material available at 10.1007/s00383-026-06527-y.

## Introduction

Major surgical procedures in the pediatric population are associated with a substantial financial burden for families, particularly in low- and middle-income countries (LMICs), where they are up to 17 times more likely to incur catastrophic expenditure [1]. Out-of-pocket expenditure includes transport to distant tertiary institutions, lost income due to time off work, and payment for food and accommodation during admission [1].

In resource-limited settings, alternatives to standard commercial stoma bags are needed to make colostomy care affordable and accessible. Published reports from LMICs describe local adaptations, such as betel leaves, cloth diapers and wrap-around waistbands as safe, inexpensive alternatives to stoma bags [2–4]. Betel leaves were estimated to cost Bangladeshi families 15 cents compared to a commercial stoma bag, which would cost US$24 per month [2]. These methods are also reported to have less odor, as they can be changed more frequently without causing skin excoriation [4]. Anyanwu et al. [3] at Minu Kano Teaching Hospital, Kano, Nigeria monitored peristomal skin after caregivers chose to manage their child’s colostomy with either a modified diaper, a wrap-around waistband, or an improvised stoma bag, using petroleum jelly as the commonly used barrier cream [3]. The authors reported that irrespective of the colostomy type, some skin excoriation was noted within the first three weeks postoperatively and then almost completely resolved by six-week follow-up [3].

This study presents an interim analysis comparing standard stoma bag care with the double-diaper technique in pediatric patients with colostomies managed at the Johannesburg Paediatric Colorectal Clinic (JPCC) at Chris Hani Baragwanath Academic Hospital, South Africa.

## Materials and methods

A prospective descriptive, contextual research design was used, and patients were randomized to either stoma bag or double-diaper care. Approval to conduct the study was obtained from the University of the Witwatersrand, Human Research Ethics Committee (Medical) (M230924).

The study was conducted within the JPCC, the Department of Pediatric Surgery, at CHBAH, a central 2 680-bed hospital in Johannesburg, South Africa. The JPCC manages more than 1 600 patients with pediatric colorectal pathology per annum. The clinic offers centralized, specialized multidisciplinary care for patients with stomas.

The study population consisted of children with new colostomies fashioned by the Department of Pediatric Surgery at CHBAH, who subsequently attended the JPCC. A convenience sampling approach was used, enrolling consecutive eligible patients whose caregivers provided written informed consent. After enrolment, patients were randomly assigned to either the stoma bag or double-diaper group using a simple randomization chart and the RedCap randomization function [5, 6]. Patients with new colostomies who attended the clinic and whose caregiver consented to participate were included in the study. Patients with ileostomies, older than three years of age, or who were attending playschools that would not accept children with a double-diaper were excluded.

No prior studies comparing peristomal skin outcomes between two colostomy-care methods in similar settings could be identified to inform the sample-size calculation. The JPCC admits between 40 and 100 new patients with stomas per year. The sample size was calculated in consultation with a biostatistician using Stata 17. A conservative minimum detectable effect size of 50%, a margin error of 15% and a 95% confidence interval was used to compute the representative sample size. This yielded a total required sample size of 86 participants, with 43 allocated to the stoma bag and 43 to the double-diaper group. The anticipated duration of the study was 36 months; an interim analysis was performed on all enrolled patients during the first 18 months to determine whether one stoma‑care method was superior and to assess whether continuing the trial was ethically justified.

**Fig. 1:**
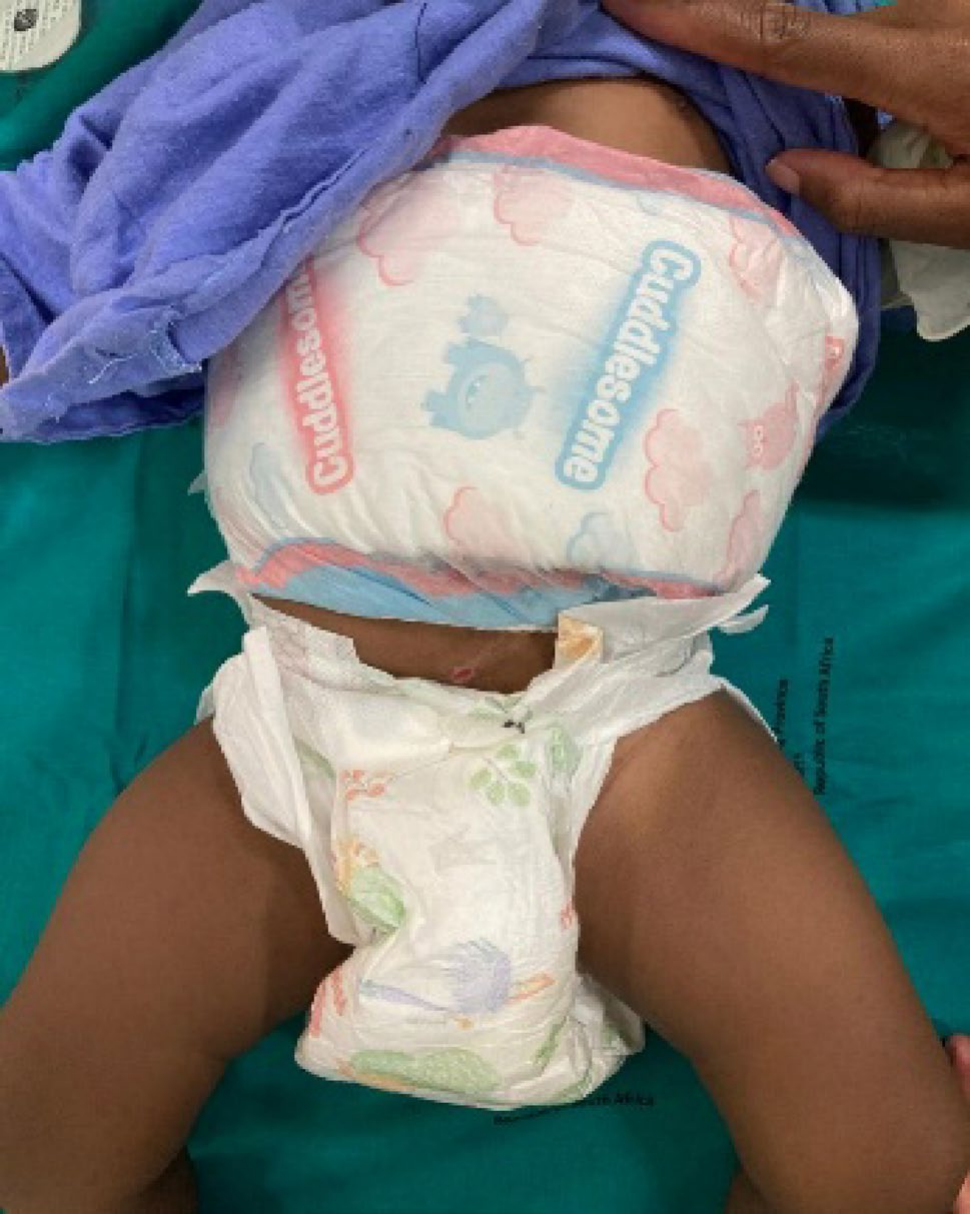
Patient with a double-diaper

The following definitions were used in the study. A double-diaper refers to one diaper applied horizontally across the abdomen covering the stoma to collect stool, and the other diaper is applied the standard way to collect urine, as shown in Fig. 1. The caregiver is the patient’s legal guardian. Skin assessment was done according to the Ostomy Skin Tool (OST) [7]. The score comprises three domains: discoloration, erosion and tissue overgrowth. Each domain is graded for affected area (0–3) and severity (0–2), with sub-scores summed to a maximum of 15, 0 reflects healthy skin and higher scores indicate more severe pathology.

The JPCC Barriers to Pediatric Colostomy Care Scoring System was used to assess challenges guardians face caring for a child with a colostomy [8].

All patients were discharged with commercial stoma bags after stoma formation and were scheduled for follow‑up at the JPCC 7 to 10 days later. At the first clinic visit, eligible patients were enrolled in the study. The colostomy was examined, and the patient was then randomly assigned to either the stoma bag or the double-diaper group using a simple randomization chart and the RedCap randomization function [5, 6].

Patients allocated to the stoma bag group received routine colostomy care with commercial stoma bags, in accordance with existing standard operating procedures. The hospital’s stoma department issued 5 to 10 stoma bags at a time, in line with hospital policy. If additional bags were required, the JPCC supplied donated stock where available, as per usual practice, and the number of extra stoma bags provided per patient was recorded. Patients attending the JPCC were not charged for stoma bags and the cost is covered by the hospital as was standard operating procedure.

Patients allocated to the double-diaper group received diapers for stoma care only, standard diapers for urine collection were for the caregivers to supply in both groups. Caregivers were provided with a single selected disposable diaper brand, with size determined by the child’s weight. For neonates, an estimated eight diapers per day were provided; for children older than one year, four diapers per day were provided for stoma care. Caregivers were provided with enough diapers, barrier cream and wipes to last until their next scheduled visit. Follow-up visits were scheduled according to the doctor’s evaluation of the patient and the stoma. Diapers and barrier crème were purchased using grant funding as the hospital does not provide diapers for out-patient use.

At every follow-up appointment, caregivers were asked whether they were satisfied with their current stoma care method. Caregivers who reported dissatisfaction were offered the option to switch to the alternative stoma‑care method. Reasons for switching were recorded, and caregivers were provided with the corresponding stoma‑care supplies until the next follow‑up visit. Patients were followed up until stoma closure.

Data was entered anonymously into an electronic REDCap database at each clinic visit. Categorical variables were summarized by frequency and percentage tabulation. Normally distributed continuous variables (age and weight) were reported as mean and standard deviation, non‑normally distributed variables (travel distance and travel cost), reported as median and interquartile range. T‑tests were used to compare continuous variables (age, weight, travel distance and travel cost). Chi‑squared tests to compare categorical variables (sex, pathology, anatomic level of colostomy, type of stoma and JPCC Barriers to Pediatric Colostomy responses) and ANOVA to compare OST skin scores [9].

**Results**.

**Fig. 2:**
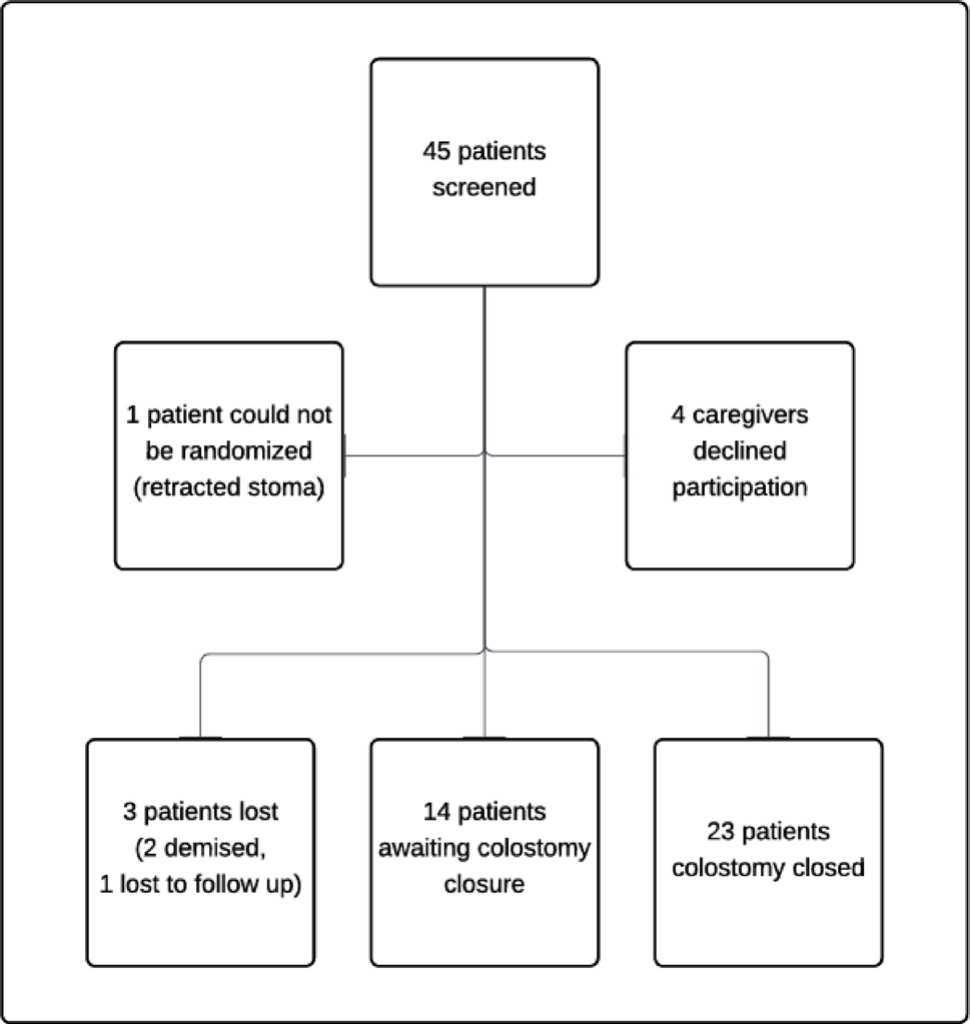
Sample realization

Over 18 months, 45 patients were screened, and the sample realization is shown in Fig. 2. Of the 40 patients who participated, 23 (57.5%) had their definitive surgical procedure and colostomy closure by the time of the study. The patient demographics are displayed in Table 1.

**Table 1:** Patient demographics

Variable	Total (40 patients)	Stoma bag (19 patients)	Double-diaper (21 patients)	
	mean (SD)	mean (SD)	mean (SD)	*p*-value
Age at enrolment (days)	110.15 (151.22)	99.95 (75.41)	119.38 (198.20)	0.68
Weight (kg)	4.57 (1.51)	4.59 (1.59)	4.55 (1.51)	0.82

Sex	*n* (%)	*n* (%)	*n* (%)	*p*-value
Male	18 (45)	9 (22.50)	9 (22.50)	0.78
Female	22 (55)	10 (25.00)	12 (30.00)	
Pathology				
Anorectal malformation	30 (75)	12 (30)	18 (45)	0.12
Hirschsprung’s disease	7 (17.50)	4 (10)	3 (7.50)	
Necrotizing enterocolitis/spontaneous intestinal perforation	3 (7.50)	3 (7.50)	–	
Colostomy type				
Divided	16 (40)	7 (17.50)	9 (22.50)	0.70
End	24 (60)	12 (30)	12 (30)	
Anatomic level of colostomy				
Ascending	1 (2.50)	1 (2.50)	–	0.42
Transverse	2 (5)	2 (5)	–	
Descending	2 (5)	1 (2.50)	1 (2.50)	
Sigmoid	29 (72.50)	13 (32.50)	16 (40)	
Not specified	6 (15)	2 (5)	4 (10)	
Caregiver employment status				
Employed	6 (15)	4 (10)	2 (5)	0.31
Unemployed	34 (85)	15 (37.50)	19 (47.50)	
Housing				
Formal	29 (72.50)	16 (40)	13 (32.50)	0.12
Informal	11 (27.50)	3 (7.50)	8 (20)	
Ablution facilities				
Inside toilet	19 (47.50)	9 (22.50)	10 (25)	0.73
Toilet outside, flushing	15 (37.50)	7 (17.50)	8 (20)	
Outside pit latrine	2 (5)	-	2 (5)	
Community toilet	4 (10)	3 (7.50)	1 (2.50)	

Social-economic factors	median (IQR)	median (IQR)	median (IQR)	*p*-value
Distance to hospital (km)	31.2 (42.30)	28.5 (38.03)	33 (40)	0.17
Travel cost (rands)	50.00 (66)	64.00 (154)	43.50 (55.25)	0.68

At enrolment 19 (47.50%) patients were randomized to the stoma bag and 21 (52.50%) to the double-diaper group. By the interim analysis 12 (63.16%) remained in the stoma bag group, 16 (76.19%) remained in the double-diaper group, and 12 (29.27%) had switched groups at least once. Of the 23 who reached stoma closure, 14 (45.00%) patients remained in their allocated group for the duration of the study. Four (21.05%) changed from stoma bag to double-diaper, three (14.29%) changed from double-diaper to stoma bag, and two (10.53%) switched groups multiple times.

**Fig. 3:**
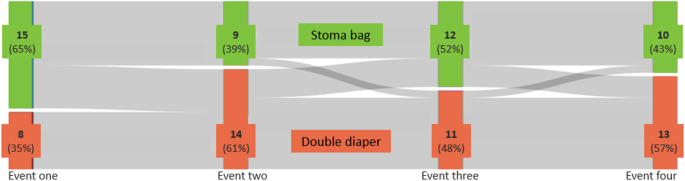
Patient flow between care groups highlighting four sentinel events for the 23 patients who had their stomas reversed

Changing groups was due to guardian requests 7 (38.89%) and 11 (61.11%) based on clinical indication. Guardians requested a change from the stoma bag group to the double-diaper group but never the other way round. Reasons included less odor, skin reaction to the stoma bag and the double-diaper being more conducive to their home environment. Some patients changed groups more than once and the reasons were recorded on each occasion. Patients were switched from the stoma bag group to the double-diaper group by the healthcare provider for skin excoriation which improved with double-diapers and then changed back to stoma bags. The flow of patients between care groups, for the 23 patients who exited the study, is summarized in Fig. 3 highlighting four sentinel events.

**Fig. 4:**
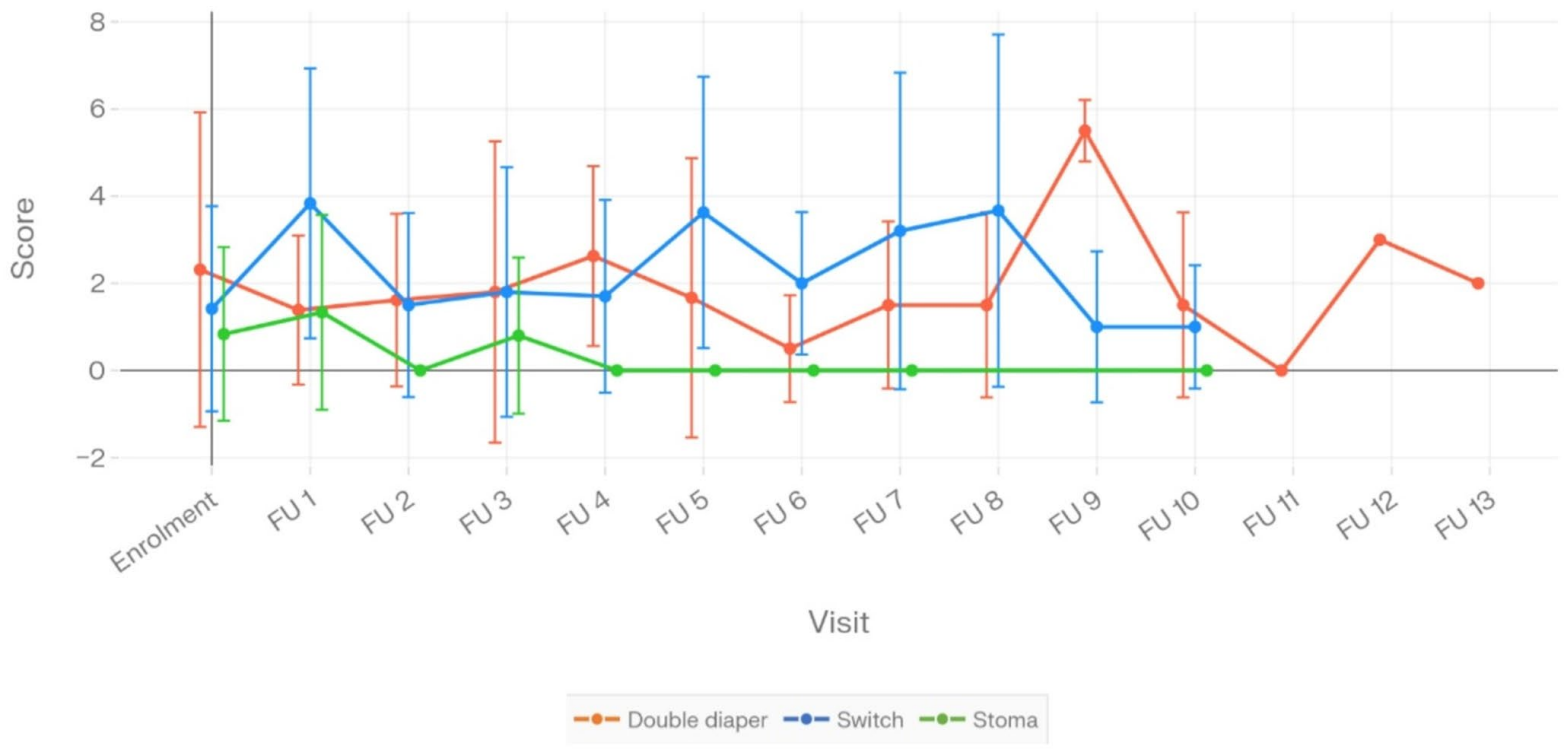
Mean OST scoring at enrollment and follow up (FU) visits for the different groups

Figure 4 shows the changes in OST scores in the different groups over the study period. Enrolment mean (SD) peristomal skin scores were 1.21 (2.27) for the stoma bag group and 1.95 (3.29) for the double-diaper group (p-value 0.26); follow-up scores among those remaining in their original groups were 0.46 (1.38) for the stoma bag group and 1.45 (2.19) for the double-diaper group (p-value 0.01). Patients who swapped groups had mean (SD) scores of 1.42 (2.35) on enrollment and 2.15 (2.65) at follow-up.

The mean (SD) number of clinic visits was 3.92 (2.47) for the stoma bag group, 5.75 (3.87) for the double-diaper group, and 7.75 (2.83) for the mixed group (p-value 0.01). At each visit, caregivers were asked if the supply of products was sufficient. When supplies were depleted, 11 (27.50%) returned for more, 29 (69.05%) purchased diapers, and 1 (2.38) caregiver used an unspecified homemade option.

The full JPCC Barriers to Pediatric Colostomy Care Scoring System results are shown in Supplementary Table 1. Twelve (80%) caregivers in the stoma group felt they received adequate supply of their respective stoma care products compared to 11 (50%) in the double-diaper group and 6 (40%) in the switch group (p-value 0.04). in the stoma bag group, 1 caregiver (6.7%) reported out-of-pocket expenditure compared to 5 (29.4%) in the double diaper group (p-value 0.01). Patients who remained in the stoma bag group reported satisfaction with bag quality 10 (66.7%), compared to those who switched group 4 (26.7%) (p-value 0.02). Five (33%) caregivers in the stoma group felt that family and friends were scared to help care for their child with a stoma compared to 14 (63.6%) in the double-diaper group and 3 (17.6%) in the switch group (p-value 0.02).

The financial implications for each group were estimated using the average number of clinic visits, travel costs and the cost of stoma-care supplies per patient. Because patients in the double‑diaper group attended more frequent follow‑up, their travel costs were higher; however, the unit cost of commercial stoma bags and ostomy rings was substantially greater than that of diapers. For the study, diapers were supplied at a bulk special rate by a manufacturer, and stomas were priced at the rate charged to government institutions. The cost of follow-up included travel and stoma supplies based on the average number of visits per group. Under these assumptions, the average per‑patient cost was R 4350.69 (USD 255.50) in the stoma bag group and R 1498,68 (USD 88.01) in the double-diaper group.

## Discussion

This was an interim analysis of the prospective study to assess if double-diaper colostomy care was a safe, cost-effective option in a setting where patient-appropriate commercial stoma bags were not always available.

The two groups were comparable in age, sex and underlying pathology, the institution’s protocol of diverting all patients with anorectal malformations with a sigmoid colostomy before delayed definitive repair, which explains why anorectal malformations and sigmoid colostomies predominated [10, 11]. Although most caregivers lived in formal housing, just under half had in‑house plumbing, and more than half relied on external ablution facilities; in this context, we speculate that caregivers at JPCC may prefer disposable single‑use double‑diapers for colostomy care because they simplify effluent disposal in settings with limited sanitation.

The majority of South Africans depend on 14-seater minibus taxis to access hospital care, and caregivers in this study travelled a median (IQR) of 31.2 (42.3) km to reach the hospital. Transporting both a baby and a monthly supply of diapers for those in the double-diaper group on crowded public transport emerged as an unanticipated logistical challenge for this study. What initially appeared to caregivers as the benefit of receiving a bulk monthly supply of diapers thus became impractical, and, to compensate, patients in the double‑diaper group required more frequent follow‑up visits (mean 5.73) than those in the stoma bag group (mean 3.92).

Despite randomization, the double-diaper group had a higher OST score at enrollment 1.95 (3.29) compared to 1.21 (2.27) for the stoma bag group, this difference was not statistically significant and may be incidental. Across follow‑up, mean OST scores in the double-diaper group remained significantly elevated compared to the stoma group (p-value 0.01) yet showed overall improvement over time. Clinically patients in the double diaper group settled over time with higher skin scores not impacting the patient or caregiver. In many patients with anorectal malformations, we observed a transient rise in OST scores around the time of their posterior sagittal ano-rectoplasty, when peri‑operative antibiotics are routinely administered; we hypothesize that antibiotic‑related changes in stool consistency through the stoma contributed to this episodic skin deterioration, typically occurring two to three months before stoma closure rather than at the end of follow‑up. In contrast, patients with necrotizing enterocolitis or Hirschsprung disease usually undergo a two‑stage pathway in which the second operation is stoma closure and thus do not experience a comparable interim peak in OST scores related to a separate definitive procedure. Taken together, these findings suggest that double‑diaper care can be used safely in this setting, although peristomal skin outcomes were, on average, less favorable than with commercial stoma bags and require closer monitoring.

Caregivers in the switch group appeared to struggle the most, with consistently higher JPCC Barriers to Pediatric Colostomy Care Scores throughout, in line with the OST results, and reflecting the fact that these patients often had more complicated stomas. In the double‑diaper group, 11 (50%) caregivers reported an inadequate supply of diapers, which contributed to unscheduled, earlier‑than‑planned clinic visits.

Caregivers in the stoma bag group reported having an adequate supply of bags 80% of the time; when they ran out, they either returned to the clinic for additional bags or used diapers they had purchased themselves until the next appointment. Caregivers in both study groups reported difficulty obtaining stoma care and support at local clinics, relying instead on the central hospital for supplies. This is largely because pediatric colostomy bags are seldom available in local facilities, are difficult to source in the community and are prohibitively expensive online.

Stoma bags appeared to make it easier for caregivers to involve family and friends in day‑to‑day care, whereas caregivers in the double‑diaper group more often reported that relatives were fearful of helping with the baby’s stoma. This reluctance likely reflects the need to expose the colostomy at every passage of stool when using double‑diapers, in contrast to stoma bags, which conceal the stoma and reduce the need for others to see or handle it directly; overall, the study underscores that families managing more complex colostomies face the greatest challenges and may require additional targeted support in already busy clinics.

There was an unanticipated caregiver preference for allocation towards, or remaining in, the double-diaper group. Gaining an honest explanation for this preference was challenging, as some caregivers feared that disclosing their motivation might jeopardize their continued access to diapers. Although neither group was charged for stoma bags or diapers within the study, we speculate that diapers may have held additional perceived value for resale or for use in the standard way, and their greater physical volume may have reinforced the sense of receiving a more substantial benefit.

As the first randomized study to compare double‑diaper and commercial stoma‑bag care in pediatric colostomy patients, this work also has important limitations. The sample size was calculated for the planned full trial rather than this 18‑month interim timepoint, so the analysis may be underpowered to detect more modest differences in skin outcomes and costs. Crossover between groups, although reflective of real‑world caregiver preferences and clinical decision‑making, complicates causal inference regarding the effect of each stoma‑care method. Implementation demanded additional administrative time at each clinic visit in an already high‑volume service with staff working at capacity and the involvement of multiple members of the multidisciplinary team, and, as a single‑center study in an urban South African tertiary hospital, the findings may not be generalizable to rural settings or health systems with different ostomy‑product supply chains. Nonetheless, the study demonstrates that teams in resource‑constrained environments can generate contextually relevant evidence to inform global pediatric stoma‑care policy and practice.

## Conclusion

In resource-constrained environments, pediatric stoma patients and their caregivers face multiple, intersecting challenges, including unemployment, long travel distance to tertiary centers and the disposal of effluents without in-house plumbing. This interim randomized analysis shows that double‑diaper care is an accessible, affordable alternative when commercial pediatric stoma bags are intermittently unavailable, but that peristomal skin outcomes are less favorable than with stoma bags and require closer monitoring. By quantifying both skin outcomes and costs, this study provides context‑specific evidence to guide colostomy‑care policy and highlights the need for additional support for families managing complex stomas in low‑resource settings.

## Supplementary Information

Below is the link to the electronic supplementary material.

Supplementary Material 1

## Data Availability

Data available on request.

## Declarations

### Trial registration number for randomized clinical trials

National Health Research Database (Reference GP_202309_016).
