# Supplementary material for: An interim analysis of the standard care (stoma bags) and double-diapers in pediatric patients with stomas at a tertiary hospital in South Africa
Source: Pediatr Surg Int. 2026 Jul 3;42(1):288. doi: 10.1007/s00383-026-06527-y (PMC13331829; doi:10.1007/s00383-026-06527-y)
Supplement: Supplementary file 1 — Supplementary Material 1 [file 383_2026_6527_MOESM1_ESM.docx]

**An interim analysis of the standard care (stoma bags) and double-diapers in pediatric patients with stomas at a tertiary hospital in South Africa**

Supplementary table 1 The JPCC Barriers to Pediatric Colostomy Care Scoring System

|  | **Question** | **Response** | **Total**  **n (%)** | **Stoma bag**  **n (%)** | **Double-diaper**  **n (%)** | **Switch**  **n (%)** | **p-value** |
| --- | --- | --- | --- | --- | --- | --- | --- |
| 1 | There are no stoma bags available at the clinic | Always | 8 (15.1) | 3 (20.0) | 3 (13.6) | 2 (12.5) | 0.52 |
|  |  | Sometimes | 4 (7.5) | 2 (13.3) |  | 2 (12.5) |  |
|  |  | Never | 31 (58.5) | 9 (60.0) | 13 (59.1) | 9 (56.2) |  |
|  |  | Not applicable | 10 (18.9) | 1 (6.7) | 6 (27.3) | 3 (18.8) |  |
| 2 | I am given enough stoma bags to last until my next visit | Always | 29 (55.8) | 12 (80.0) | 11 (50.0) | 6 (40.0) | 0.04 |
|  |  | Sometimes | 6 (11.5) | 2 (13.3) | 1 (4.5) | 3 (20.0) |  |
|  |  | Never | 6 (11.5) |  | 2 (9.1) | 4 (26.7) |  |
|  |  | Not applicable | 11 (21.2) | 1 (6.7) | 8 (36.4) | 2 (13.3) |  |
| 3 | The stoma bags leak or do not stick properly | Always | 9 (17.3) |  | 4 (18.2) | 5 (33.3) | 0.02 |
|  |  | Sometimes | 11 (21.2) | 4 (26.7) | 3 (13.6) | 4 (26.7) |  |
|  |  | Never | 20 (38.5) | 10 (66.7) | 6 (27.3) | 4 (26.7) |  |
|  |  | Not applicable | 12 (23.1) | 1 (6.7) | 9 (40.9) | 2 (13.3) |  |
| 4 | I am given the wrong type of stoma bag for my child. (e.g. a bag with a tap instead of a colostomy bag) | Always | 3 (5.7) | 1 (6.7) |  | 2 (12.5) | 0.05 |
|  |  | Never | 36 (67.9) | 13 (86.7) | 12 (54.5) | 11 (68.8) |  |
|  |  | Not applicable | 14 (26.4) | 1 (6.7) | 10 (45.5) | 3 (18.8) |  |
| 5 | I am given the wrong size stoma bags for my child | Always | 4 (7.5) | 1 (6.7) | 1 (4.5) | 2 (12.5) | 0.10 |
|  |  | Sometimes | 2 (3.8) |  | 2 (9.1) |  |  |
|  |  | Never | 34 (64.2) | 13 (86.7) | 10 (45.5) | 11 (68.8) |  |
|  |  | Not applicable | 13 (24.5) | 1 (6.7) | 9 (40.9) | 3 (18.8) |  |
| 6 | I am not given enough stoma care products (e.g. barrier seals or protective skin sprays) | Always | 18 (33.3) | 5 (33.3%) | 7 (31.8) | 6 (35.3) | 0.87 |
|  |  | Sometimes | 3 (5.6) |  | 1 (4.5) | 2 (11.8) |  |
|  |  | Never | 22 (40.7) | 7 (46.7%) | 9 (40.9) | 6 (35.3) |  |
|  |  | Not applicable | 11 (20.4) | 3 (20.0%) | 5 (22.7) | 3 (17.6) |  |
| 7 | I know where to get stoma care products | Always | 37 (68.5) | 11 (73.3) | 15 (68.2) | 11 (64.7) | 0.81 |
|  |  | Never | 14 (25.9) | 4 (26.7) | 5 (22.7) | 5 (29.4) |  |
|  |  | Not applicable | 3 (5.) |  | 2 (9.1) | 1 (5.9) |  |
| 8 | I am scared that I will hurt my child when caring for their stoma. | Always | 6 (11.1) | 1 (6.7) | 3 (13.6) | 2 (11.8) | 0.83 |
|  |  | Sometimes | 14 (25.9) | 3 (20.0) | 7 (31.8) | 4 (23.5) |  |
|  |  | Never | 34 (63.0) | 11 (73.3) | 12 (54.5) | 11 (64.7) |  |
| 9 | My family and friends are scared to help care for my child with a stoma. | Always | 22 (40.7) | 5 (33.3) | 14 (63.6) | 3 (17.6%) | 0.02 |
|  |  | Sometimes | 5 (9.3) | 1 (6.7) |  | 4 (23.5%) |  |
|  |  | Never | 24 (44.4) | 7 (46.7) | 8 (36.4) | 9 (52.9) |  |
|  |  | Not applicable | 3 (5.6) | 2 (13.3) |  | 1 (5.9) |  |
| 10 | I am worried my child's stoma bag will leak or show in public. | Always | 10 (18.5) | 5 (33.3) | 2 (9.1) | 3 (17.6) | 0.26 |
|  |  | Sometimes | 6 (11.1) | 3 (20.0) | 1 (4.5) | 2 (11.8) |  |
|  |  | Never | 33 (61.1) | 7 (46.7) | 16 (72.7) | 10 (58.8) |  |
|  |  | Not applicable | 5 (9.3) |  | 3 (13.6) | 2 (11.8) |  |
| 11 | My child sometimes pulls their stoma bag off. | Always | 2 (3.7) | 1 (6.7) |  | 1 (5.9) | 0.18 |
|  |  | Sometimes | 20 (37.0) | 7 (46.7) | 8 (36.4) | 5 (29.4) |  |
|  |  | Never | 23 (42.6) | 7 (46.7) | 7 (31.8) | 9 (52.9) |  |
|  |  | Not applicable | 9 (16.7) |  | 7 (31.8) | 2 (11.8) |  |
| 12 | I find it difficult to know if there are problems with my child's stoma and how to handle the problems | Always | 5 (9.3) | 1 (6.7) | 2 (9.1) | 2 (11.8) | 0.90 |
|  |  | Sometimes | 17 (31.5) | 6 (40.0) | 7 (31.8) | 4 (23.5) |  |
|  |  | Never | 32 (59.3) | 8 (53.3) | 13 (59.1) | 11 (64.7) |  |
| 13 | It is a lot of work to care for my child's stoma | Always | 10 (18.5) | 5 (33.3) | 4 (18.2) | 1 (5.9) | 0.16 |
|  |  | Sometimes | 8 (14.8) |  | 4 (18.2) | 4 (23.5) |  |
|  |  | Never | 36 (66.7) | 10 (66.7) | 14 (63.6) | 12 (70.6) |  |
| 14 | I battle to talk to or understand the doctors and nurses because of language difficulties | Always | 6 (11.1) | 1 (6.7) | 3 (13.6) | 2 (11.8) | 0.81 |
|  |  | Sometimes | 9 (16.7) | 2 (13.3) | 5 (22.7) | 2 (11.8) |  |
|  |  | Never | 39 (72.2) | 12 (80.0) | 14 (63.6) | 13 (76.5) |  |
| 15 | My child's stoma makes their development delayed. For example, when they start crawling or walking. | Always | 5 (9.3) |  | 4 (18.2) | 1 (5.9) | 0.23 |
|  |  | Sometimes | 5 (9.3) | 3 (20.0) | 1 (4.5) | 1 (5.9) |  |
|  |  | Never | 29 (53.7) | 6 (40.0) | 12 (54.5) | 11 (64.7) |  |
|  |  | Not applicable | 15 (27.8) | 6 (40.0) | 5 (22.7) | 4 (23.5) |  |
| 16 | I have access to water or electricity | Always | 42 (77.8) | 14 (93.3) | 17 (77.3) | 11 (64.7) | 0.15 |
|  |  | Sometimes | 10 (18.5) | 1 (6.7) | 5 (22.7) | 4 (23.5) |  |
|  |  | Never | 2 (3.7) |  |  | 2 (11.8) |  |
| 17 | It is difficult to carry my child because of their stoma | Always | 3 (5.6) | 2 (13.3) | 1 (4.5) |  | 0.30 |
|  |  | Sometimes | 1 (1.9) |  |  | 1 (5.9) |  |
|  |  | Never | 50 (92.6) | 13 (86.7) | 21 (95.5) | 16 (94.1) |  |
| 18 | Is it difficult to see the stoma (part of the intestine) outside my child's skin. | Always | 9 (16.7) | 4 (26.7) | 3 (13.6) | 2 (11.8) | 0.80 |
|  |  | Sometimes | 3 (5.6) | 1 (6.7) | 1 (4.5) | 1 (5.9) |  |
|  |  | Never | 42 (77.8) | 10 (66.7) | 18 (81.8) | 14 (82.4) |  |
| 19 | There is a long waiting list at the hospital to close my child's stoma | Always | 3 (5.6) |  | 3 (13.6) |  | 0.43 |
|  |  | Sometimes | 3 (5.6) | 1 (6.7) | 1 (4.5) | 1 (5.9) |  |
|  |  | Never | 34 (63.0) | 11 (73.3) | 11 (50.0) | 12 (70.6) |  |
|  |  | Not applicable | 14 (25.9) | 3 (20.0) | 7 (31.8) | 4 (23.5) |  |
| 20 | My child is too shy to play with other children (reluctant) | Always | 2 (3.7) | 1 (6.7) | 1 (4.5) |  | 0.60 |
|  |  | Sometimes | 1 (1.9) |  |  | 1 (5.9) |  |
|  |  | Never | 22 (40.7) | 7 (46.7) | 10 (45.5) | 5 (29.4) |  |
|  |  | Not applicable | 29 (53.7) | 7 (46.7) | 11 (50.0) | 11 (64.7) |  |
| 21 | I am careful about my child playing with other children | Always | 19 (35.2) | 7 (46.7) | 7 (31.8) | 5 (29.4) | 0.70 |
|  |  | Sometimes | 9 (16.7) | 3 (20.0) | 3 (13.6) | 3 (17.6) |  |
|  |  | Never | 7 (13.0) | 2 (13.3) | 4 (18.2) | 1 (5.9) |  |
|  |  | Not applicable | 19 (35.2) | 3 (20.0) | 8 (36.4) | 8 (47.1) |  |
| 22 | People discriminate against my child. (e.g. they cannot attend creche, school or play sport) | Always | 4 (7.4) | 2 (13.3) | 1 (4.5) | 1 (5.9) | 0.76 |
|  |  | Sometimes | 6 (11.1) | 2 (13.3) | 3 (13.6) | 1 (5.9) |  |
|  |  | Never | 23 (42.6) | 6 (40.0) | 11 (50.0) | 6 (35.3) |  |
|  |  | Not applicable | 21 (38.9) | 5 (33.3) | 7 (31.8) | 9 (52.9) |  |
| 23 | I am comfortable talking to anyone outside the clinic about having a child with a stoma | Always | 36 (66.7) | 7 (46.7) | 16 (72.7) | 13 (76.5) | 0.09 |
|  |  | Sometimes | 2 (3.7) |  | 2 (9.1) |  |  |
|  |  | Never | 15 (27.8) | 8 (53.3) | 3 (13.6) | 4 (23.5) |  |
|  |  | Not applicable | 1 (1.9) |  | 1 (4.5) |  |  |
| 24 | I received adequate stoma care training. | Always | 48 (88.9) | 14 (93.3) | 19 (86.4) | 15 (88.2) | 0.14 |
|  |  | Sometimes | 2 (3.7) |  |  | 2 (11.8) |  |
|  |  | Never | 4 (7.4) | 1 (6.7) | 3 (13.6) |  |  |
| 25 | I was made aware of the social challenges of having a child with a stoma. | Always | 27 (50.0) | 7 (46.7) | 7 (31.8) | 13 (76.5) | 0.07 |
|  |  | Sometimes | 5 (9.3) | 2 (13.3) | 2 (9.1) | 1 (5.9) |  |
|  |  | Never | 22 (40.7) | 6 (40.0) | 13 (59.1) | 3 (17.6) |  |
| 26 | The nurses working in the clinic know about stoma care | Always | 52 (96.3) | 15 (100.0) | 21 (95.5) | 16 (94.1) | 0.45 |
|  |  | Sometimes | 1 (1.9) |  | 1 (4.5) |  |  |
|  |  | Never | 1 (1.9) |  |  | 1 (5.9) |  |
| 27 | If my child is admitted to the hospital for any reason, the ward nurses know about stoma care | Always | 45 (83.3) | 12 (80.0) | 18 (81.8) | 15 (88.2) | 0.55 |
|  |  | Sometimes | 1 (1.9) |  | 1 (4.5) |  |  |
|  |  | Never | 7 (13.0) | 3 (20.0) | 3 (13.6) | 1 (5.9) |  |
|  |  | Not applicable | 1 (1.9) |  |  | 1 (5.9) |  |
| 28 | The doctors working in the clinic know about stoma care. | Always | 51 (96.2) | 15 (100.0) | 22 (100.0) | 14 (87.5) | 0.31 |
|  |  | Sometimes | 1 (1.9) |  |  | 1 (6.2) |  |
|  |  | Never | 1 (1.9) |  |  | 1 (6.2) |  |
| 29 | If my child is admitted to the hospital for any reason, the ward doctors know about stoma care | Always | 46 (85.2) | 12 (80.0) | 18 (81.8) | 16 (94.1) | 0.45 |
|  |  | Sometimes | 4 (7.4) | 1 (6.7) | 3 (13.6) |  |  |
|  |  | Not applicable | 4 (7.4) | 2 (13.3) | 1 (4.5) | 1 (5.9) |  |
| 30 | I sometimes receive different or confusing stoma care advice from health care workers | Always | 2 (3.7) |  | 1 (4.5) | 1 (5.9) | 0.85 |
|  |  | Sometimes | 11 (20.4) | 4 (26.7) | 4 (18.2) | 3 (17.6) |  |
|  |  | Never | 40 (74.1) | 11 (73.3) | 16 (72.7) | 13 (76.5) |  |
|  |  | Not applicable | 1 (1.9) |  | 1 (4.5) |  |  |
| 31 | I have stoma care at my local hospital and I don't need to travel to a big (academic) hospital | Always | 13 (24.1) | 5 (33.3) | 4 (18.2) | 4 (23.5) | 0.72 |
|  |  | Sometimes | 1 (1.9) |  | 1 (4.5) |  |  |
|  |  | Never | 37 (68.5) | 10 (66.7) | 15 (68.2) | 12 (70.6) |  |
|  |  | Not applicable | 3 (5.6) |  | 2 (9.1) | 1 (5.9) |  |
| 32 | It is expensive to travel to the hospital or clinic for stoma care | Always | 24 (44.4) | 4 (26.7) | 10 (45.5) | 10 (58.8) | 0.40 |
|  |  | Sometimes | 13 (24.1) | 5 (33.3) | 6 (27.3) | 2 (11.8) |  |
|  |  | Never | 17 (31.5) | 6 (40.0) | 6 (27.3) | 5 (29.4) |  |
| 33 | I have to spend extra money on stoma care products when I run out of product | Always | 10 (18.5) | 1 (6.7) | 4 (18.2) | 5 (29.4) | 0.01 |
|  |  | Sometimes | 15 (27.8) | 1 (6.7) | 6 (27.3) | 8 (47.1) |  |
|  |  | Never | 29 (53.7) | 13 (86.7) | 12 (54.5) | 4 (23.5) |  |
| 34 | I am unable to work because of my child's stoma | Always | 27 (50.0) | 9 (60.0) | 10 (45.5) | 8 (47.1) | 0.82 |
|  |  | Sometimes | 5 (9.3) | 1 (6.7) | 3 (13.6) | 1 (5.9) |  |
|  |  | Never | 22 (40.7) | 5 (33.3) | 9 (40.9) | 8 (47.1) |  |
| 35 | I sometimes miss clinic appointments because I do not have money to travel | Always | 1 (1.9) |  | 1 (4.8) |  | 0.74 |
|  |  | Sometimes | 13 (24.5) | 3 (20.0) | 5 (23.8) | 5 (29.4) |  |
|  |  | Never | 39 (73.6) | 12 (80.0) | 15 (71.4) | 12 (70.6) |  |
